# Miscellany

**Published:** 1906-04

**Authors:** 


### ITEMS.

---

The second hand, Harvard operating chairs advertised for sale in this issue by Mr. L. D. Platt, No. 1050 Ellicott Spuare, are as good as new. Mr. Platt is the Buffalo representative of the W. D. Allison concern of Indianapolis, and of the Wagner Electrical Co., of Chicago.

---

Mr. Wm. T. Collins, whose card appears in the Journal, has been in the medical book business in Buffalo for the past thirty years. He can obtain any medical book published, on short notice. See him for medical books.

